# BoPEP4, a C-Terminally Encoded Plant Elicitor Peptide from Broccoli, Plays a Role in Salinity Stress Tolerance

**DOI:** 10.3390/ijms23063090

**Published:** 2022-03-13

**Authors:** Anyi Wang, Jingsong Guo, Sibo Wang, Ying Zhang, Fangfang Lu, Jingbin Duan, Zhao Liu, Wei Ji

**Affiliations:** Department of Biotechnology, College of Life Science, Northeast Agricultural University, Harbin 150030, China; s200901097@neau.edu.cn (A.W.); jingsongguo@neau.edu.cn (J.G.); b210901003@neau.edu.cn (S.W.); zy275205@163.com (Y.Z.); s200902006@neau.edu.cn (F.L.); S200902018@neau.edu.cn (J.D.); liuzhao_liuzhao@yeah.net (Z.L.)

**Keywords:** broccoli, plant elicitor peptide (Pep), salinity stress tolerance, RNA-seq, KEGG pathway enrichment

## Abstract

Plant peptide hormones play various roles in plant development, pathogen defense and abiotic stress tolerance. Plant elicitor peptides (Peps) are a type of damage-associated molecular pattern (DAMP) derived from precursor protein PROPEPs. In this study, we identified nine *PROPEP* genes in the broccoli genome. qRT-PCR analysis indicated that the expression levels of *BoPROPEPs* were induced by NaCl, ABA, heat, SA and *P. syringae* DC3000 treatments. In order to study the functions of Peps in salinity stress response, we synthesized BoPep4 peptide, the precursor gene of which, *BoPROPEP4*, was significantly responsive to NaCl treatment, and carried out a salinity stress assay by exogenous application of BoPep4 in broccoli sprouts. The results showed that the application of 100 nM BoPep4 enhanced tolerance to 200 mM NaCl in broccoli by reducing the Na^+^/K^+^ ratio and promoting accumulation of wax and cutin in leaves. Further RNA-seq analysis identified 663 differentially expressed genes (DGEs) under combined treatment with BoPep4 and NaCl compared with NaCl treatment, as well as 1776 genes differentially expressed specifically upon BoPep4 and NaCl treatment. GO and KEGG analyses of these DEGs indicated that most genes were enriched in auxin and ABA signal transduction, as well as wax and cutin biosynthesis. Collectively, this study shows that there was crosstalk between peptide hormone BoPep4 signaling and some well-established signaling pathways under salinity stress in broccoli sprouts, which implies an essential function of BoPep4 in salinity stress defense.

## 1. Introduction

As sessile organisms that lack opportunities to avoid environmental stress conditions, plants need to continuously recognize and respond to changing environments for survival. Numerous studies have shown that there are various molecular mechanisms regulating plant stress responses, such as stress signal perception, calcium signaling, reactive oxygen species (ROS) burst and protein kinase signaling [1,2]. In particular, 5 to 60 amino acid long peptides characteristic of plant hormones have emerged as essential mediators of cell-to-cell and long-distance signaling during plant growth and stress responses [3].Approximately 210 small coding genes are annotated as precursor genes of hormone-like peptides in *Arabidopsis*, which are classified into more than 20 families [4]. Each class of peptide phytohormones plays versatile roles in various aspects of plant growth, development, pathogen defense, and abiotic stress response [3].

Plant elicitor peptides (Peps), well-characterized members of the damage-associated molecular patterns (DAMPs) family, were demonstrated to enter into the extracellular space when a cell is attacked by pathogens or destroyed by injury, inducing pattern-triggered immunity (PTI) defense processes [5]. Peps are derived from C-terminal regions of precursor protein PROPEPs, which are processed by Ca^2+^-dependent type-II metacaspases (MCs) [6,7]. There are eight Peps (Pep1-Pep8) originating from precursor proteins PROPEP1 to PROPEP8 in *Arabidopsis*, which are recognized by two homologous leucine-rich repeat receptor kinases, PEPR1 and PEPR2 [8]. Among them, AtPep1 and AtPep2 were identified as the key mediators of plant innate immunity [5], whereas AtPep3 has been shown to be essential for the induction of salinity stress tolerance, as well as immune response in plants [9]. However, the downstream molecular signaling of salinity tolerance mediated by AtPep3-PEPR1 is not defined because none of the typical abiotic stress-responsive genes or genes related to other phytohormones had been identified in either *AtPROPEP3* overexpression or synthetic AtPep3 peptide-treated plants [9]. In *Arabidopsis*, it is indicated that AtPeps trigger stomatal closure in a PEPR-dependent manner by activating guard-cell anion channels [10]. In addition to the well-studied immunity responses mediated by Peps in leaf tissues, several recent studies focused on the roles of Peps in roots. Pep1-PEPR2 was evidenced to regulate the abundance and distribution of the auxin efflux carriers PIN-FORMED2 (PIN2) and PIN3, leading to altered local auxin accumulation and inhibition of root growth in *Arabidopsis* [11]. Further studies illustrated that Pep1 regulated root growth by affecting ROS production [12], as well as promoting proton extrusion into apoplast in an AHA2-dependent manner [13], which has shed light on the signaling crosstalk between Pep1 and some recognized pathways. Besides studies in model plant *Arabidopsis*, pretreatment with Peps has also been identified to protect maize and rice plants against herbivore attack by activation of mitogen-activated protein kinase signaling and generation of reactive oxygen species (ROS) and defense metabolites [14,15]. A recent study demonstrated that OsPep3 conferred rice resistance to brown planthopper by regulating jasmonic acid biosynthesis, as well as lipid and phenylpropanoid metabolism through transcriptomics and metabolomics profiling [16]. Moreover, the presence and pathogen defense activity of Peps within Rosaceae was characterized in [17]. *Prunus persica* peptides PpPep1 and PpPep2 were proved to cause PTI-like transcriptome reprogramming at nanomolar doses in vivo [18]. Interestingly, GmPep914, a novel peptide isolated from soybean leaves, was capable of alkalinizing the media of soybean suspension cells and inducing the expression of genes involved in pathogen defense and phytoalexin production [19]. Taken together, it can be speculated that Peps are conserved regulators of immune responses across plant species that also contribute to salt stress tolerance; however, the principles by which Peps enhance plant tolerance to salt still need to be investigated.

Broccoli (*Brassica oleracea* var. *italica*) is a cruciferous vegetable crop species consumed worldwide. However, environmental stresses, such as salinity, severely affect the growth and productivity of broccoli seedlings, and to the best of our knowledge, little is known about the functions of peptide hormones in broccoli. Therefore, in this study, we aim to investigate how broccoli copes with salinity stress regulated by peptide hormone Peps, as well as the underlying mechanisms. To this end, we identified nine precursor genes of Peps in broccoli and investigated the expression patterns of *BoPROPEPs* under abiotic stress treatments. Through exogenous application of synthetic BoPep4 peptide in broccoli seedlings based on liquid culture salinity stress assay, the functional role of BoPep4 was measured. The molecular mechanism of salinity tolerance regulated by BoPep4 peptide was further studied by subsequent RNA-seq analysis. Our findings suggest new insights into Pep signaling mechanisms controlling the salinity defense through fine-tuned modulation of phytohormone metabolism, as well as accumulation of wax and cutin biosynthesis.

## 2. Results

### 2.1. Identification and Expression Patterns of BoPROPEP Genes in Broccoli

**Figure 1:**
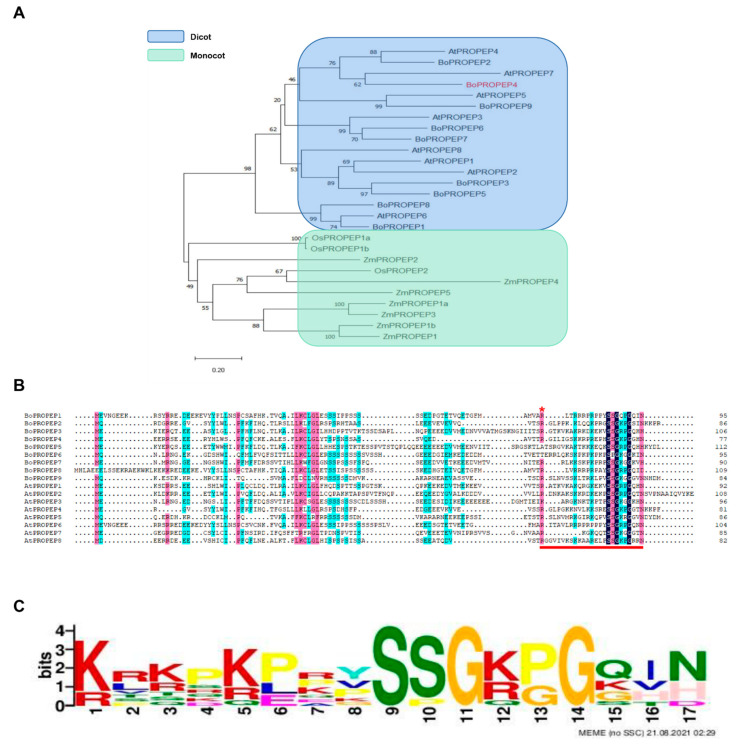
Identification and sequence analysis of BoPROPEP genes in broccoli. (**A**) Full-length amino-acid sequences of identified BoPROPEPs and published PROPEPs from other species were used to build a bootstrapped neighbor-joining tree. Major families are highlighted with colors according to the legend. BoPROPEP4 (red highlight) was mainly studied in this research. Scale-bar: amino-acid substitutions per site. (**B**) Alignment of nine BoPROPEPs and eight AtPROPEPs reveals conserved and divergent residues. Identical and similar residues are shaded in black, blue and pink. The conserved arginine (*) preceding the Pep sequence (indicated by the red line on the bottom) is highlighted with a red asterisk. (**C**) Depiction of the consensus sequences of aligned Pep sequences in broccoli using MEME tools.

In order to identify *PROPEP* genes in broccoli, a tblastn search (E values < 1 × 10^−5^) was carried out in the broccoli genome database using the amino acid sequences of published AtPROPEP1-8 in *Arabidopsis* as queries. A total of nine putative *BoPROPEP* genes were identified in the broccoli genome, which were distributed unevenly throughout the genome, designated as *BoPROPEP1* to *BoPROPEP9* based on their chromosome positions (Table S1 and Figure S1). The phylogenetic tree of identified BoPROPEPs and the published PROPEPs in other species is shown in Figure 1A, indicating that these PROPEPs were separated into plant-family-specific clusters. For example, BoPROPEP4 shows more similarity to AtPROPEP7 than to other orthologues of distantly related plant species. Although there were small sequence identities among these PROPEP orthologues, even within the family of a single species, they were predicted to be post-translationally processed and releasing 23-residue mature peptide (Figure 1B) and sharing the conserved Pep motif SSG-x2-G-x2-N at the C terminus (Figure 1C). 

**Figure 2:**
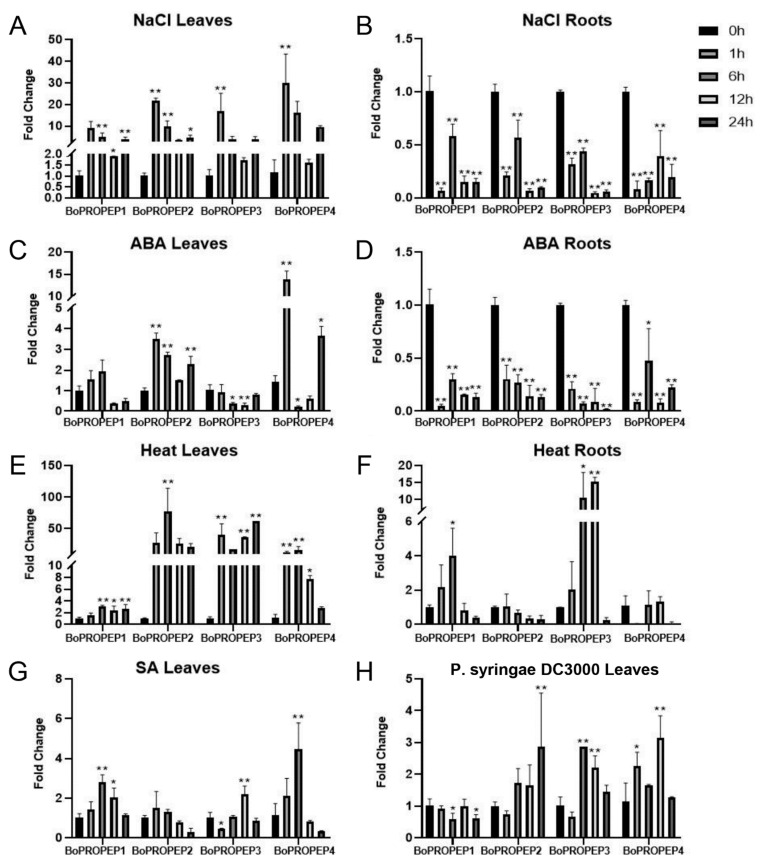
Relative expression of *BoPROPEP* genes at 0, 1, 6, 12 and 24 h after different treatments. Changes in mRNA levels of *BoPROPEP1*, *BoPROPEP2*, *BoPROPEP3* and *BoPROPEP4* genes with stress treatments in leaves and roots were determined by quantitative real-time PCR. (**A**,**B**) NaCl (100 mM); (**C**,**D**) heat stress (37 °C); (**E**,**F**) ABA (5 µM); (**G**) SA (2 mM); (**H**) *P. syringae* DC3000 (OD600 = 0.02) The expression of each gene under the control treatment was set as 1. Changes in transcript abundance are represented as fold change by calibrating the relative mRNA levels of each time point with the relative mRNA levels of the 0 h time point. Error bars represent the standard deviation (SD) of three biological repeats. Asterisks indicate that the differences between the control and treatments were significant (* 0.01 < *p* < 0.05, ** *p* < 0.01).

Furthermore, a tblastn (E-values < 1 × 10^−5^) search was performed of the previously published broccoli transcriptome data [20] to obtain a preliminary understanding of the tissue-specific expression pattern of *BoPROPEP* genes in broccoli seedlings. As a result, only four members, *BoPROPEP1* to *BoPROPEP4*, were retrieved during seed germination and sprout development (Table S2), probably due to the low expression levels of other *BoPROPEP* genes. In order to get an idea of the transcriptional regulations of *BoPROPEPs* under stress, we explored the expression changes of *BoPROPEP1* to *BoPROPEP4* in 11-day-old broccoli leaves and roots in response to NaCl (100 mM), heat (37 °C), ABA (5 µM), SA (2 mM) and *P. syringae* DC3000 (OD600 = 0.02) treatments. Quantitative-PCR analyses revealed that *BoPROPEP1* to *BoPROPEP4* were all significantly upregulated under salinity conditions in broccoli leaves (Figure 2A). These four genes exhibited similar expression patterns at sequential time points and reached the maximum expression levels after 1 h of salt treatment. Notably, *BoPROPEP4* was most strongly upregulated among these four genes and was induced over 30-fold more than that in leaves before treatment. Similarly, the expression of *BoPROPEP4* was induced strikingly in response to ABA treatment in leaves and peaked at 1 h after stress induction, then declined during the later treatment and returned to be upregulated after 24 h of treatment (Figure 2C). The results indicate that *BoPROPEP4* showed early and rapid responses to salinity and ABA. However, this was not the case for expression of *BoPROPEPs* in roots, which decreased to very low levels during exposure to salt and ABA treatments (Figure 2B,D). When treated with heat stress, expression of these four genes were upregulated in broccoli leaves (Figure 2E), whereas only *BoPROPEP1* and *BoPROPEP3* were found to be induced in response to heat in roots (Figure 2F). Since the contributions of PROPEPs in plant defense responses have been extensively studied, we also investigated the expression levels of *BoPROPEPs* under SA and *P. syringae* DC3000 treatments. The results showed that the transcriptional levels of *BoPROPEP1*, *BoPROPEP2* and *BoPROPEP4* were induced when treated with SA (Figure 2G). In addition, *BoPROPEP2*, *BoPROPEP3* and *BoPROPEP4* were slightly upregulated under *P. syringae* DC3000 treatment (Figure 2H). These results indicated that *BoPROPEPs* might play important roles in response to ABA-mediated salinity stress besides pathogen defense. Therefore, BoPep4 derived from *BoPROPEP4* was selected for further investigation due to the highly inducible expression of the precursor gene under ABA and salt stress.

### 2.2. Exogenous Application of BoPep4 Alleviated Salinity-Induced Stress in Broccoli Seedlings

**Figure 3:**
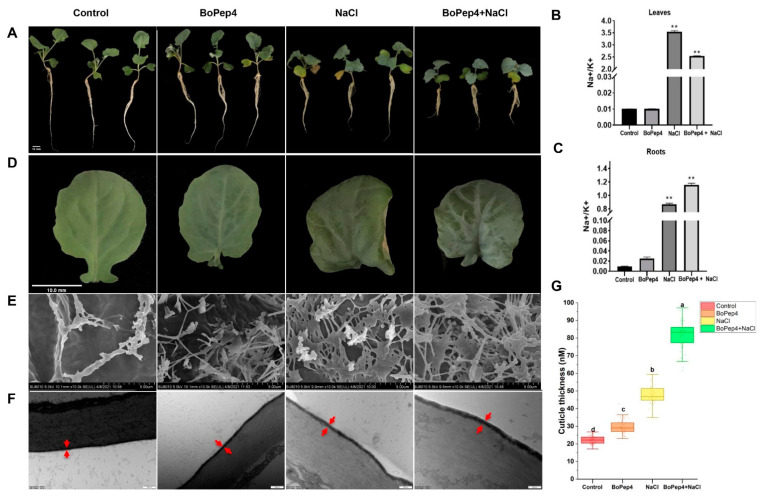
BoPep4 peptide application enhanced salinity stress tolerance in broccoli seedlings. (**A**) Phenotype of broccoli seedlings after 7 days of exposure to 100 nM BoPep4, 200 mM NaCl and pretreatment with 100 nM BoPep4 peptide for 3 days followed by combined treatment with 100 nM peptide plus 200 mM NaCl for 7 days; bar = 1 cm. (**B**) Na^+^/K^+^ in leaves under different treatments. (**C**) Na^+^/K^+^ in roots under different treatments. (** *p* < 0.01) (**D**) Broccoli leaves under different treatments. (**E**) Scanning electron microscopy (SCM) images of cuticular wax crystals on the leaves of broccoli under 100 nM BoPep4, 200 mM NaCl and combination treatments. (**F**) Transmission electron microscope (TEM) images (red arrow) and (**G**) thickness measurements of the cutin in the leaves of broccoli under 100 nM BoPep4, 200 mM NaCl and combination treatments. Values are the averages of three replicates ± SD. Different letters represent significant differences according to Duncan’s multiple range tests (*p* < 0.05).

To investigate the function of BoPep4 in plants under salinity stress, mature BoPep4 peptide (GILIGSKKRPREPHSSGKPGGHN) was synthesized (Figure S2). In order to evaluate the effects of BoPep4 application in salinity tolerance of broccoli seedlings, it was necessary to determine the appropriate initial concentrations of NaCl and BoPep4 peptide. Given that broccoli is moderately sensitive to salinity, different concentrations of NaCl (0, 100, 150, 200 and 300 mM) were applied to 11-day-old hydroponic broccoli sprouts for 7 days. The results indicated that the plant growth was similar to that of control plants when the supplemented NaCl was lower than 150 mM; when the NaCl concentration was increased to 200 mM, the leaves showed salt-induced chlorosis, which was further verified by DAB staining, quantitatively measured MDA contents and chlorophyll contents (Figure S3). Moreover, in order to check the dose effect of BoPep4 peptide on the growth of broccoli plants, we treated the 3-day-old broccoli seedlings with different concentrations of the BoPep4 peptide (0, 10, 50, 100, 200 and 500 nM) separately. The results indicated that application of BoPep4 peptide inhibited root growth of broccoli seedlings in a dose-dependent manner, as reflected by significantly shortened roots under 50 nM of BoPep4 treatment compared with the control but less root growth inhibition when the BoPep4 concentration was achieved at 100 nM (Figure S4). Thus, 100 nM BoPep4 pretreatment before 200 mM NaCl and 100 nM BoPep4 combination treatments to the hydroponically grown broccoli seedlings was conducted in further experiments. After hydroponic growth for 7 days, broccoli seedlings treated with 100 nM of BoPep4 and 200 mM NaCl exhibited shorter roots but dark green leaves compared with the seedlings with withered and yellow leaves subjected to salt stress (Figure 3A,D). After 200 mM NaCl treatment, the Na^+^/K^+^ ratios in both leaves and roots of broccoli plants were increased significantly compared with the control and treatment with 100 nM of BoPep4 (Figure 3B,C). However, when exposed to a combination of BoPep4 and NaCl, the broccoli leaves exhibited contrasting trends in roots and had a lower Na^+^/K^+^ ratio than in plants under NaCl treatment (Figure 3B), indicating that the application of BoPep4 alleviated the severe ionic imbalance caused by salinity in broccoli leaves.

Interestingly, the broccoli leaves under different treatments presented divergent surface morphologies (Figure 3D). Thus, the deposition of epicuticular wax crystals on the leaf surface of broccoli seedlings under BoPep4, NaCl and the combination treatments was examined. Relative to the mock control, broccoli leaves displayed more epicuticular wax crystals with a scale- and rod-like shape under BoPep4 treatment, whereas the density of wax crystals was higher on leaves under NaCl treatment, and these wax crystals tended to have a plate-type shape (Figure 3E). In particular, the density of wax crystals was much higher on the leaf surface of seedlings under BoPep4 and NaCl combination treatment than those under NaCl treatment, creating a dense covering with an intricate dendritic rodlet shape (Figure 3E). Furthermore, TEM analysis of leaf epidermal cells revealed that the thickness of the cutin was increased by approximately 1.8-fold in broccoli seedlings under BoPep4 and NaCl combined treatment compared with broccoli under NaCl treatment (Figure 3F,G).

### 2.3. Identification of Differentially Expressed Genes Regulated by BoPep4 in Response to NaCl Stress

**Figure 4:**
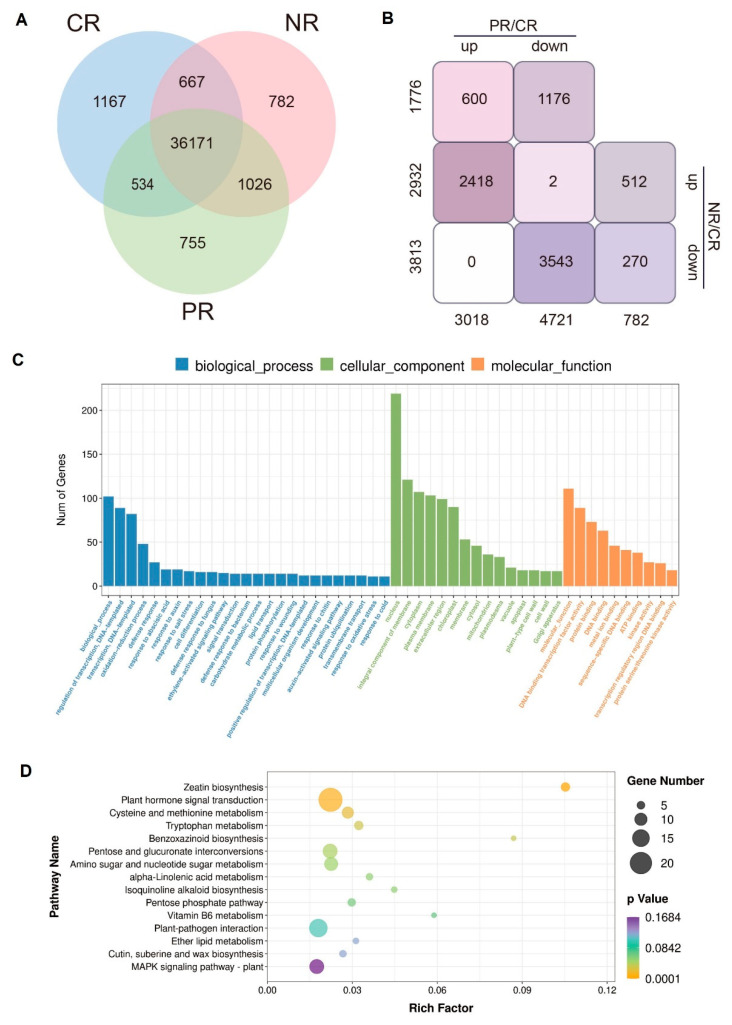
RNA sequencing analyses of broccoli seedlings with different treatments. (**A**) Venn diagram of the number of genes in CR, PR and NR samples. (**B**) Number of differentially expressed genes (DEGs) between PR, NR and CR obtained by RNA-Seq. (**C**) GO analysis classified the DEGs into three groups (molecular function, biological process and cellular component). (**D**) KEGG categories of the DEG analysis; the x-axis represents the enrichment ratio, the y-axis represents the KEGG pathways, and bubble’s size indicates the number of genes annotated to a certain KEGG pathway.

To further elucidate the molecular basis by which BoPep4 induced salinity tolerance of broccoli seedlings, we conducted comparative transcriptome analysis based on Illumina sequencing technology. Nine libraries were created and sequenced from three biological replicates for control roots (CR), roots treated with 200 mM NaCl (NR) and roots treated with a combination of 100 nM BoPep4 and 200 mM NaCl (PR). After quality control and filtering for each sample, more than 38 million clean reads were provided, and >89% of these reads could be mapped to the reference genome (Table S3). The total number of genes identified in each sample is shown in Figure 4A. The differences in the expression levels between samples are log2-transformed ratios. Further comparisons of the RNA-Seq data among the CR, NR, and PR plants (*p* value < 0.05) revealed 8521 differentially expressed genes (DEGs) (Table S4). Compared with the CR, 2932 upregulated and 3813 downregulated genes were found in the NR samples, whereas 3018 upregulated and 4721 downregulated genes were found in the PR samples (Figure 4B). Interestingly, only two genes were regulated in opposite manners between the NR vs. CR group and the PR vs. CR group (Figure 4B). In addition, 600 upregulated and 1176 downregulated genes were specifically identified in the PR vs. CR group, but none existed in the NR vs. CR group (Figure 4B). Moreover, compared with NR, there were 186 genes upregulated and 477 genes downregulated in PR samples (Table S4). Therefore, we chose these 663 DEGs in the PR vs. NR group and 1776 specific DEGs in the PR vs. CR group for GO and KEGG pathway enrichment analyses to study the transcriptomic mechanism underlying the salt tolerance of broccoli seedlings regulated by BoPep4. Both groups of DEGs presented similar GO-term enrichment related to regulation of transcription, oxidation-reduction process and response to stress in the biological process category. In the cellular component category, most DEGs were enriched in the nucleus, followed by the integral component of the membrane. Among the molecular function categories, both groups of DEGs were enriched in DNA-binding transcription factor activity, protein binding and DNA binding, as well as kinase activity (Figure 4C and Figure S5A). In KEGG pathway enrichment analysis, the main enriched DEGs were involved in phytohormone-signaling transduction and plant–pathogen interaction. Moreover, both groups of DEGs shared common pathways pertaining to cutin, suberine and wax biosynthesis (Figure 4D and Figure S5B).

### 2.4. BoPep4 Influences Auxin Homeostasis during Salinity Stress

**Figure 5:**
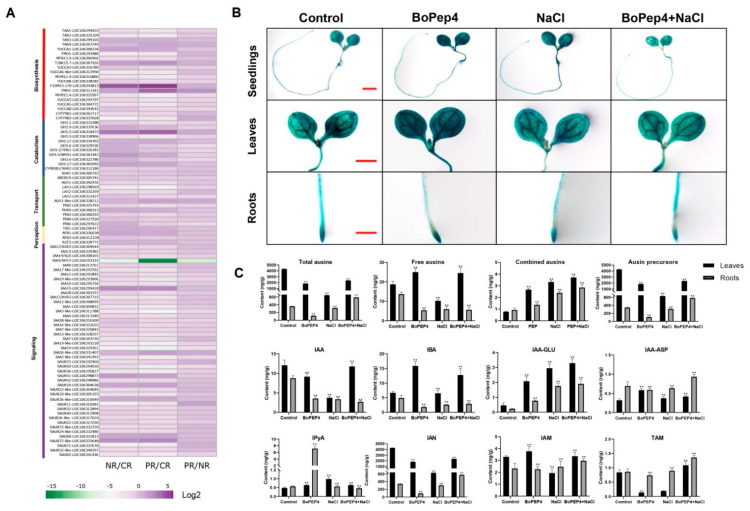
BoPEP4 treatment alters auxin accumulation under salt stress. (**A**) The expression of the genes associated with auxin biosynthesis, metabolism, transport, perception, and signaling in CR, PR and NR analyzed by RNA sequencing. The detailed expression data are available in Table S5. (**B**) Histochemical staining for GUS activity of *Arabidopsis* seedlings transformed with *DR5::GUS* under 50 nM BoPep4, 150 mM NaCl and combination treatments. (**C**) Auxin contents of broccoli leaves and roots under 100 nM BoPep4, 200 mM NaCl and combination treatments. Three independent experiments were performed. Error bars indicate SD (** *p* < 0.01).

Following analysis of KEGG pathway enrichment of DEGs, we observed that the pathway “plant hormone signal transduction” was most overrepresented (Figure 4D), and considering research in *Arabidopsis* demonstrated that Pep1 induced root hair formation and root growth inhibition by auxin redistribution [11], we first explored the expression profiles of genes within the auxin homeostasis and signaling pathways (Figure 5A and Table S5). The RNA-seq data showed that the expression levels of TAA1/TAR and YUCCA families, as well as CYP79B2/3, were differentially regulated in NR/CR, PR/CR and PR/NR, suggesting that BoPep4 application may affect the biosynthesis of indole-3-acetic acid (IAA) under NaCl treatment. Besides, the expression levels of auxin conjugase GH3 genes and auxin transporters AUX1 and PINs were altered both in NR/CR and PR/CR, indicating that salinity and BoPep4 treatments may affect auxin catabolism and polar auxin transport, which may lead to changed auxin accumulation. We also observed that a majority of auxin-responsive genes were differentially expressed after salinity and the combination treatments. It is noteworthy that the degree of differential expression of genes in PR/CR was more intense than in NR/CR, implying exogenous application of BoPep4 may regulate auxin homeostasis to mediate salt stress response. 

To confirm this, we investigated auxin localization in response to BoPep4, NaCl and combination treatment using *DR5::GUS* transgenic *Arabidopsis* plants. The results showed that β-glucuronidase (GUS) staining was reduced in roots under the treatment conditions compared with the control plants, but exogenous BoPep4 increased root GUS activity in DR5-GUS transgenic plants under salinity stress (Figure 5B). Similarly, the application of 50 nM BoPep4 increased GUS staining in leaves of plants under salinity stress (Figure 5B). Furthermore, auxin contents were measured in broccoli seedlings under 100 nM BoPep4, 200 mM NaCl and combination treatments (Figure 5C). Compared to the control, the total auxin contents in leaves and roots decreased dramatically under NaCl treatment, with depressed free auxins and auxin precursors but increased auxin conjugates. When only treated with BoPep4, the total auxin contents in leaves and roots were lower than in the mock control but much higher than in plants under salt treatment. In particular, BoPep4 treatment resulted in significant enhancement of free auxin content in leaves and indole-3-pyruvic acid (IPyA) content in roots compared with plants under control and salt treatment, indicating that the IPyA auxin biosynthetic pathway is operative in response to BoPep4. Furthermore, pretreatment with 100 nM BoPep4 significantly alleviated salt-induced depression of auxin contents, which was consistent with the RNA-seq data and GUS activity. These results suggest that BoPpe4 may have a positive effect on auxin accumulation in plants’ adaptation to salinity stress. 

### 2.5. BoPep4 Accentuated ABA Response under Salinity Stress 

**Figure 6:**
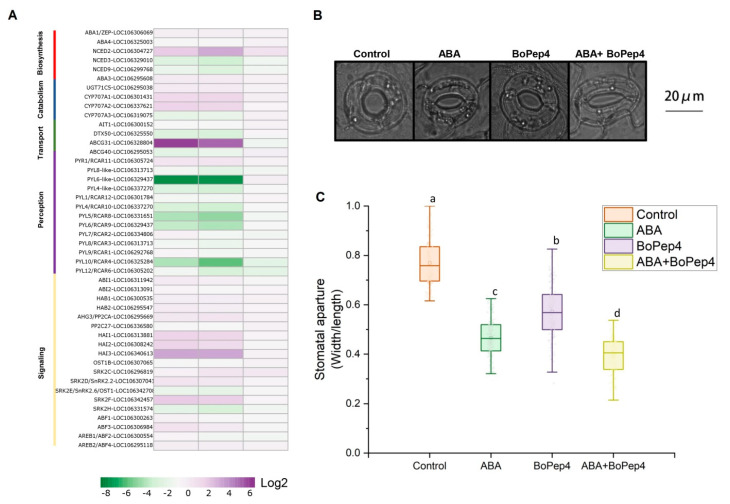
BoPEP4 treatment alters ABA response under salt stress. (**A**) The expression of the genes associated with ABA biosynthesis, metabolism, transport, perception and signaling in CR, PR and NR analyzed by RNA sequencing. The detailed expression data are available in Table S6. (**B**) Representative images of guard cells of broccoli treated with ABA, PEP and ABA + PEP. Bars = 20 μm. (**C**) Stomatal aperture (the ratio of stomatal width to length) measurements of guard cells of broccoli treated with ABA, PEP and ABA + PEP. In each experiment, 150 stomata from separate seedlings were measured. Three independent experiments were performed. Different letters represent significant differences according to Duncan’s multiple range tests (*p* < 0.05).

ABA plays an essential role in salinity stress response and is always considered as a stress hormone [21]. We found that genes involved in ABA biosynthesis were differentially regulated by treatments with the most obviously induced expression of NINE-CIS-EPOXYCAROTENOID DIOXYGENASE 2 (NCED2) under the combination of BoPep4 and NaCl (Figure 6A and Table S6). We also detected the expression of genes involved in ABA catabolism, such as CYP707A and UDP glucosyltransferases UGT71C5. Differences in their expression levels in NaCl treatment and the combination of BoPep4 and NaCl were not obvious, indicating less possible effect of BoPep4 on ABA metabolism under NaCl stress in broccoli roots. Moreover, ABCG31 was significantly induced by NaCl and the combination treatments, suggesting BoPep4 promoted the transport of ABA under salt stress through activation of ABA transporter genes. Besides, ABA receptor family PYL/RCARs were downregulated in response to BoPep4 treatment under salt treatment, thus altering the expressions of ABA signaling members. To further illustrate the effects of BoPep4 on the ABA response, stomatal movements of *Arabidopsis* leaves were measured under BoPep4, ABA or the combination treatments. Leaf samples were first incubated in stomatal opening solution under light to facilitate the full opening of stomata. Compared with the dramatic stomatal closure under ABA treatment, the stomatal aperture was slightly larger in response to BoPep4 treatment (Figure 6B,C). Intriguingly, the stomatal aperture was much smaller when exposed to the combination treatment than ABA or BoPep4 separate treatment (Figure 6B,C), indicating the ABA response was enhanced by the application of BoPep4. 

### 2.6. BoPep4 Enhances Wax and Cutin Accumulation under Salinity Stress

**Figure 7:**
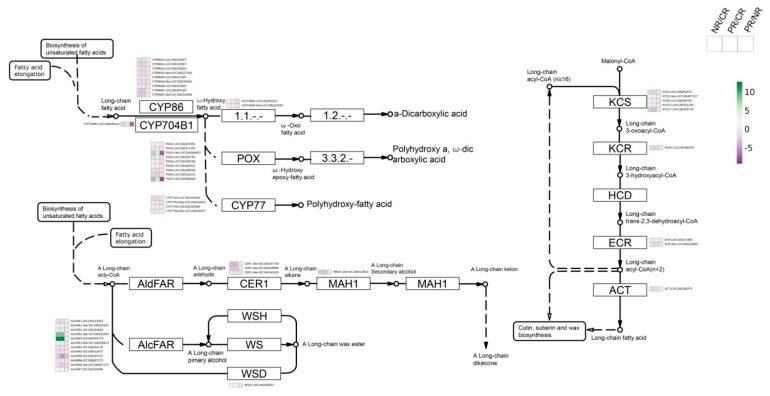
BoPEP4 treatment enhances wax and cutin accumulation under salt stress. Expression of the genes associated with cuticular wax biosynthesis. Detailed expression data are available in Table S7.

KEGG analysis showed that some DEGs were enriched in the “cutin, suberine and wax biosynthesis” pathway (Figure 4D). Cuticular waxes, consisting of very long-chain fatty acids (VLCFAs) and their derivatives, were reported to play an essential role in protecting plants against environmental stresses [22]. DEGs involved in fatty acid elongation, alkane biosynthesis, and secondary alcohol and ketone biosynthesis pathways were identified (Figure 7 and Table S7). Alcohol-forming fatty acyl reductases (AlcFARs) that function as NADPH-dependent alcohol-forming reductases in biosynthesis of fatty alcohols exhibited significant upregulation under the combination treatment of BoPep4 and NaCl. Moreover, KCS, KCR, ECR and ACT involved in VLCFA biosynthesis showed differential expression under BoPep4 and NaCl treatments. These differentially expressed genes may contribute to the much heavier deposition of epicuticular wax crystals and thicker cutin layer of broccoli leaves treated by BoPep4 in response to NaCl treatment (Figure 3E–G). Together, these results indicate that BoPep4 application enhanced the salinity resistance of broccoli sprouts by regulating epidermal wax and cutin synthesis.

## 3. Discussion

Peps are a recently discovered class of peptide hormone that have been extensively reported to function as inducers of pattern-triggered immunity and amplifiers of the immune response against diverse pathogens and insect herbivores in *Arabidopsis*, rice and maize [5,8,14,16,23]. Peps are cleaved and released from larger precursor proteins called PROPEPs. A comprehensive search for PROPEPs throughout dicotyledonous plants in Brassicaceae, Solanaceae and Rosaceae, as well as monocotyledon crops in Poaceae revealed extensive differences in their amino acid sequences [17,24]. In this study, nine *BoPROPEPs* were identified in the broccoli genome, and the amino acid-based homology was very low (Figure 1A). However, the family-specific Pep motifs critical for Pep activity were conservative (Figure 1B,C), which were supposed to recognize and activate the receptor PEPRs [24,25]. 

The spatial and temporal expression profiles of *PROPEP* genes in *Arabidopsis* have been investigated extensively and displayed complex regulation patterns, which indicates various biological functions. *PROPEP3* has been shown to respond to infection with fungal pathogens and was highly induced by salt stress, whereas expression levels of *PROPEP1* and *PROPEP2* were identified to be enhanced upon treatments with fungal, bacterial pathogens, herbivores and wounding [9,23,26,27]. In this study, we observed the complex regulation of *BoPROPEPs* in broccoli leaves and roots in response to different stimuli (Figure 2). Here, in agreement with studies in other species, we found transcriptional levels of *BoPROPEP3* and *BoPROPEP4* rose upon both *P. syringae* DC3000 and SA treatments, which indicates the involvement of both genes in pathogen defense. Given the crosstalk between immune receptors and signaling regulators under abiotic stress [28], we detected the transcriptional responses of *BoPROPEPs* under salt, ABA and heat treatments. Consistent with our expectations, these four *BoPROPEPs* showed elevated transcription as early as 1 h upon NaCl treatment and exhibited continuous high expression levels 24 h after salinity stress in leaves. Similar upregulation of *BoPROPEPs* was found in leaves under ABA and heat treatments. However, induction of *BoPROPEPs* under salinity and ABA treatments was not detected in roots. Our observations confirm the previous finding in *Arabidopsis* that promoters of *PROPEP1* and *PROPEP3* were strongly activated in response to NaCl treatment in leaves [29]. Notably, *BoPROPEP4* showed the highest expression levels under salinity and ABA treatment among these four paralogs; thus, *BoPROPEP4* was selected in the follow-up study. 

A previous study demonstrated that chemically synthesized AtPep1 was as active as native AtPep1 [5]. In particular, enhancement of salinity tolerance in *Arabidopsis* was conferred by both AtPep3 peptide treatment and overexpression of the *AtPROPEP3* gene [9]. These results prompted us to synthesize the deduced mature BoPep4 peptide and test the effect on broccoli seedlings to decipher the potential roles of BoPep4 in conferring salinity stress tolerance. It has been previously reported that salinity tolerance varied in different broccoli cultivars at different developmental stages [30]. In the present study, the 11-day-old broccoli (cv. Youxiu) sprouts showed severe salt damage when the NaCl concentration was increased to 200 mM (Figure S3). Similarly to previous reports [18], we found BoPep4 treatment triggered significant seedling growth inhibition in a concentration-dependent manner, with the minimum inhibition achieved at 100 nM doses (Figure S4). Thus, 100 nM BoPep4 pretreatment followed by 200 mM NaCl treatment was selected for further experiments. Our experiments demonstrated that exogenous BoPep4 application can significantly enhance salt tolerance in broccoli seedlings by inhibiting Na^+^ uptake in leaves (Figure 3A,B), as well as enhanced wax and cutin accumulation (Figure 3E–G). Collectively, we detected BoPep4-mediated enhanced NaCl tolerance in broccoli, suggesting a systemic response to the BoPep4 root treatment, which is in line with the finding that local application of Pep in *Arabidopsis* leaves triggered systemic immune signaling [31]. However, the mechanisms underlying salinity tolerance in broccoli conferred by peptide hormone BoPep4 remains poorly understood. Recent findings indicated rapid activation of the salt-responsive transcriptome in *Arabidopsis* was responsive to pattern recognition [32]. Here, we analyzed the broccoli response to salinity stress under exogenous application of broccoli Pep through RNA-Seq, which allowed us to characterize the transcriptomics information that would facilitate understanding the molecular mechanisms of BoPep4-induced salinity tolerance. 

We observed extensive transcriptional reprogramming of broccoli roots under salinity treatment (NR), as well as combination treatment of BoPep4 with NaCl (PR) compared with the mock control (CR) (Figure 4 and Table S4). There were a variety of DEGs enriched in “plant–pathogen interaction” and “MAPK signaling” pathways (Figure 4), which is consistent with the view established in *Arabidopsis* and peach that Pep induced major regulation in activation of pattern-triggered immunity (PTI) signaling [5,18]. Pep perception contributing to defense responses depends on the corresponding receptor PEPRs in *Arabidopsis* [8]. In this study, we detected the upregulation of the Pep receptor genes *BoPEPR1* and *BoPEPR2* in the PR/NR group (Table S4). Moreover, botrytis-induced kinase1 (BIK1) and PBS1-like1 (PBL1) were demonstrated to interact with PEPR1 to regulate Pep1-induced defenses in response to pathogens and herbivores [32,33,34,35,36]. Similarly to *BoPEPRs*, enhanced expression levels of *BoBIK1* and *BoPBL1* were detected in broccoli roots under combination treatment of NaCl and BoPep4 (Table S4). This points towards their roles in driving Pep signal transduction in response to salinity stress in broccoli similar to that depicted in plant immunity [37].

It is well known that plant hormones play a critical role in the entire plant’s growth period, especially when surviving in an adverse environment. Pep-induced systemic immune response was described to be related to ethylene (ET), salicylic acid (SA) and jasmonic acid (JA) signal transduction [16,18,31,38]. In the current study, DEGs related to hormone signaling, with special significance of auxin and ABA hormone signal transduction, were detected. Salt-inhibited root growth was reported to be associated with reduced auxin accumulation, which was related to altered localization of the auxin transporters AUX1 and PIN1/2 [21]. Interestingly, Pep1-induced root inhibition was mimicked by exogenous auxin application, which resulted from regulation of the distribution and abundance of auxin efflux carriers PIN2 and PIN3 [11]. The downregulation of *PIN* genes resulting from salinity stress had been reported previously, which may be related to the expression of auxin-responsive *DR5::GUS* in *Arabidopsis* root [39]. Here, we found downregulation of genes related to auxin transport in NR/CR, PR/CR and PR/NR (Figure 5A and Table S5), coincidental with reduced auxin accumulation in roots under treatments (Figure 5B,C). Besides, the RNA-seq results showed that several genes involved in auxin biosynthesis were differentially regulated by BoPep4 application, which led to a significant increase in IAA and IBA contents in leaves under salt stress upon exogenous application of BoPep4 (Figure 5A,C). IAA, the main auxin in plants, is primarily synthesized from L-tryptophan (Trp) through tryptophan aminotransferases TAA/TAR. The intermediate product, IPyA, is then converted into IAA by the flavin monooxygenases YUC [40]. Notably, BoPep4 treatment remarkably enhanced the content of IPyA in broccoli roots compared with plants subjected to salinity and the mock control (Figure 5C), suggesting the activation of IAA biosynthesis by the IPyA pathway besides the major IAOx pathway. In addition to the free form, auxin conjugates were thought to play important roles in abiotic stress responses as storage forms for the active IAA. Overexpression of auxin conjugating enzymes encoded by *GH3* gene reduces auxin levels in *Arabidopsis* and causes a dwarfed phenotype [41]. On the other hand, the *GH3* knockout mutant in *Physcomitrium patens* was tolerant to 250 mM NaCl treatment [42]. In this study, the expression levels of GH3 family members were reduced in PR/NR, which may contribute to maintenance of auxin homeostasis regulated by BoPep4 under salinity stress (Figure 5A and Table S5). Based on these results, we speculated that BoPep4 may help to regulate auxin accumulation to mediate broccoli’s tolerance to salinity stress.

ABA, as one of the key stress-response phytohormones, plays an indispensable role in salt stress response. Under salinity stress, endogenous ABA levels increase rapidly, and ABA signaling is immediately activated, leading to stomatal closure and whole-plant responses [43]. In broccoli roots, BoPep4 application induced upregulation of ABA biosynthetic gene *NCED2* under salt stress (Figure 6A and Table S6), which was supposed to be correlated with increased level of ABA. Under stressed conditions, ABA functions in plants by binding the receptor PYLs, which interact with clade A PP2Cs and then release the inhibition of SnRK2s. Highly ABA-induced (HAI) 1/2/3 belonging to clade A PP2Cs were reported to function as negative regulators of stress responses [43]. The *HAI* gene was induced by ABA and wounding, and although the *hai-1* mutant displayed ABA hyposensitivity phenotype [44], *HAI 1/2/3* double and triple mutants had post-germination ABA hypersensitivity and showed increased proline and osmoregulatory solute contents at low water potential [45]. Enhanced expression levels of *HAI 1/2/3* genes were observed in PR/CR (Figure 6A), suggesting the participation of BoPep4 in regulation of ABA signaling. However, whether regulation of *HAI 1/2/3* by BoPep4 results in salt stress resistance is yet to be confirmed. In the present study, we observed an obvious decrease in stomatal aperture in response to ABA compared to the mock control and even more aggravated levels of stomatal closure upon combination treatment of ABA and BoPep4 (Figure 6B,C). Thus, it can be hypothesized that BoPep4 has a synergistic effect on ABA-induced stomata closure.

Cutin, the major component of the cuticle, together with cuticular waxes deposited outside of the cutin and within the cutin matrix, function as the hydrophobic barrier in preventing water loss to protect plants from abiotic stresses [46]. Cutin is the lipid polymer consisting of oxygenated fatty acids with a chain length of C16-C18, and cuticular waxes are composed of complex mixtures of VLCFAs, aldehydes, alkanes, secondary alcohols, ketones and other derivatives. Cuticular wax biosynthesis is known to be a complex process involving C16-C18 fatty acids synthesis, C16-C18 fatty acid elongation, and subsequent modification via either the decarbonylation or the acyl reduction pathway [47]. In our study, DEGs involved in cutin and suberin biosynthesis, wax biosynthesis and fatty acid elongation pathways were enriched in NR/CR and PR/CR (Figure 7 and Table S7). 3-Ketoacyl-CoA synthase (KCS) was proved to be a key enzyme in the fatty acid extension pathway involved in the synthesis of VLCFA wax precursors. Overexpression of *KCS* genes in *Arabidopsis* and *Brassica napus* led to increased cuticular wax deposition and improved abiotic stress tolerance [48,49]. Several members of the KCS family were identified as differentially expressed in the NR/CR and PR/CR groups, indicating their contribution to biosynthesis of the critical fatty acid elongation in broccoli sprouts under salinity stress. Fatty acyl-CoA reductase catalyzes the production of primary alcohols in the biosynthesis of cuticular wax in plants [50]. Various NADH-dependent fatty acyl reductases (FARs) have been identified and characterized in *Arabidopsis*, wheat and other species. Heterologous expression of FARs in yeast resulted in the accumulation of C24:0 and C26:0 primary alcohols, indicating the catalysis activities for suberin-associated primary alcohol formation [50,51]. According to our RNA-seq data, several alcohol-forming fatty acyl reductases (AlcFARs) showed significant upregulation under salt stress and BoPep4 treatment (Table S7). Notably, AlcFAR7 was specifically recognized in the PR/CR group, suggesting a role in cuticular wax accumulation under salinity stress regulated by BoPep4. The notion that BoPep4 application regulated the “cutin, suberine and wax biosynthesis” pathway in broccoli was further supported by SEM and TEM analyses. In accordance with the RNA-seq results, NaCl and BoPep4 treatments resulted in a higher density of wax crystals and a thicker cutin layer on the leaf surface compared with the mock control, whereas the densest array of epicuticular wax crystals consisting of longitudinal bundles of rodlets and horizontal, reticulate platelets intertwined together, as well as the thickest cutin layer, were detected on the leaf surface of seedlings under BoPep4 and NaCl combination treatment (Figure 3E–G). Taken together, it can be speculated that BoPep4 modified the cutin and wax accumulation in defense against salinity stress in broccoli sprouts. However, further in-depth studies using BoPep4 overexpression or gene-editing mutants are required to explore the detailed mechanisms of improvement of salt tolerance.

## 4. Materials and Methods

### 4.1. Identification and Characterization of BoPROPEP Genes

The sequences of PROPEP1-8 in *Arabidopsis* [26] were used to search the broccoli genome in the NCBI database (https://www.ncbi.nlm.nih.gov/genome/?term=txid3712[orgn] (accessed on 18 September 2019)) by tblastn (E values < 1 × 10^−5^). A phylogenetic tree was constructed using the neighbor-joining method using MEGA 6.0 software with 1000 bootstrap replicates to evaluate the reliability of the phylogenetic grouping. The tree files were viewed and edited using MEGA 6.0. The amino acid sequences of AtPROPEPs and BoPROPEPs were aligned using Clustal W with default parameters. The conserved motifs in BoPROPEPs genes were predicted using online MEME tools (http://meme-suite.org/tools/meme (accessed on 21 August 2021)). 

### 4.2. Plant Growth and Treatments

Broccoli (*Brassica oleracea* var. *italic* cv Youxiu) seeds were germinated on 1/2 MS (Murashige and Skoog) agar medium (pH 5.8). Ten-day-old seedlings were transferred into 1/2 MS liquid medium. For salt, ABA and SA treatments, the roots of broccoli plants were immersed in nutrient solution containing 100 mM NaCl, 5 µM ABA and 2 mM SA, respectively, at room temperature; for heat treatment, hydroponic seedlings were transferred into a 37 °C growth chamber; for pathogen treatment, the *Pseudomonas syringae* pv. tomato DC3000 (*P. syringae* DC3000) was cultured to OD600 = 0.02 as described in [20] and sprayed on the broccoli leaves until saturated. Leaf and root tissues were harvested after 0, 1, 6, 12 and 24 h exposure to each treatment. Three independent sets of control and stress treatment samples were collected, and all the samples were immediately frozen in liquid nitrogen and stored at −80 °C until use. 

### 4.3. RNA Extraction and qRT-PCR Analysis

Total RNA was extracted and treated with DNaseI using an Ultrapure RNA kit (CWBIO, Taizhou, China), and cDNA was synthesized using a cDNA Synthesis SuperMix kit (TransGen, China) following the manufacturer’s instructions. Gene-specific primers for quantitative real-time RT-PCR (qRT-PCR) analysis were designed using Primer 5.0 (Table S8). *BoACTIN-2* was used as internal reference gene. qRT-PCR reaction was performed using 2 × SYBR Green qPCR mix (SparkJade, China) with a QuantStudio TM 3 real-time PCR instrument (Thermo Fisher, Waltham, MA, USA). The PCR reaction was carried out with the following reaction conditions: 94 °C for 30 s; followed by 40 cycles of 94 °C for 5 s, 58 °C for 30 s and 72 °C for 30 s. Samples for qRT-PCR were run in 3 biological replicates with 3 technical replicates, and the data are represented as the mean ± SD (*n* = 3) for Student’s *t*-test analysis. The relative gene expression was calculated using the ΔΔCt algorithm, as in our previous study [52].

### 4.4. Peptide Synthesis and Treatment Assay

BoPep4 peptide was designed and synthesized by GeneCreate Biological Engineering Co., Ltd. (Wuhan, China) (Figure S2). The synthetic peptide was dissolved in dimethyl sulfoxide (DMSO) and diluted to 1 mM stock solution. Eight-day-old broccoli seedlings geminated on 1/2 MS agar medium were transferred to the 1/2 MS liquid for 3 days. The 11-day-old broccoli sprouts were immersed in 1/2 MS solution containing 100 nM BoPep4 and grown for 3 days to serve as the peptide pretreatment. Subsequently, 200 mM NaCl and 100 nM BoPep4 were administered at the same time in a liquid culture-based assay, which was served as BoPep4 + NaCl treatment. Salinity stress test was performed by transferring the seedlings to the liquid 1/2 MS medium for 6 days, followed by adding 200 mM NaCl into 1/2 MS medium. BoPep4 treatment was performed by replacing the liquid medium with new medium containing 100 nM BoPep4. The seedlings grown in 1/2 MS liquid medium were used as the mock control. After each treatment for 7 d, three independent sets of control and treated broccoli roots and true leaves were collected, and each replicate represented a pooled sample of three individual plants. These samples were used for Na^+^ and K^+^ contents, scanning and transmission electron microscopy and RNA-seq analyses.

### 4.5. Measurement of Na^+^ and K^+^ Contents

A volume of 0.5 g of each root and leaf sample was dried, ground into powder and digested with concentrated HNO_3_ at 110 °C for 2 h. Na^+^ and K^+^ contents were measured using an atomic absorption spectrophotometer as described previously [53].

### 4.6. Scanning and Transmission Electron Microscopy Analyses

Scanning electron microscopy (SEM) and transmission electron microscopy (TEM) were performed as previously described [54]. Briefly, the broccoli leaf blades excised from seedlings under different treatments were fixed, dehydrated, dried and coated with platinum particles using a sputter coater and examined by an SU-8010scanning electron microscopy (HITACHI). For TEM, fixed and dehydrated leaf segments were embedded in Spurr’s epoxy resin for 2 d. Ultrathin sections (80 nm) were cut using RMC PowerTome ultramicrotome. The sections were stained with uranyl acetate and lead citrate solution and observed by TEM (H-7650; Hitachi, Tokyo, Japan).

### 4.7. Transcriptome Sequencing and Data Analysis

The root samples of broccoli seedlings treated with 200 mM NaCl and 100 nM BoPep4 combined with 200 mM NaCl for 7 days were collected for transcriptomic sequencing. Broccoli seedlings grown in MS liquid were used as controls, and three biological replicates were set. Total RNA was extracted using TRIzol reagent (Invitrogen, Carlsbad, CA, USA) following the manufacturer’s procedure. The RNA amount and purity of each sample were quantified using a NanoDrop ND-1000 (NanoDrop, Wilmington, DE, USA). RNA integrity was assessed by a Bioanalyzer 2100 (Agilent, CA, USA) with RIN number >7.0 and 1 μg total RNA for cDNA library preparation. High-throughput sequencing was then performed on an Illumina Novaseq™ 6000 (LC-Bio Technology CO., Ltd., Hangzhou, China) following the recommended protocol.

Raw data were processed using cutadapt software (https://cutadapt.readthedocs.io/en/stable/ (accessed on 12 February 2021)) to remove the reads containing adaptor contamination. Then, the clean reads were mapped to the genome using HISAT2 software (https://daehwankimlab.github.io/hisat2/ (accessed on 12 February 2021)) and assembled using StringTie (http://ccb.jhu.edu/software/stringtie/ (accessed on 12 February 2021)) with default parameters. Gene expression levels were calculated using FPKM (FPKM = [total_exon_fragments/mapped_reads (millions) × exon_length (kB)]). Differentially expressed genes (DEGs) were selected with fold change >2 or fold change <0.5 and *p* value < 0.05 by DESeq2 (http://www.bioconductor.org/packages/release/bioc/html/DESeq2.html (accessed on 12 February 2021)). Gene ontology (GO) enrichment analysis of DEGs was performed by GOseq R packages based on Wallenius non-central hypergeometric distribution. Pathways associated with DEGs were determined using the Kyoto Encyclopedia of Genes and Genomes (KEGG, http://www.kegg.jp (accessed on 12 February 2021)). KOBAS 2.0 (http://kobas.cbi.pku.edu.cn/download.do (accessed on 12 February 2021)) software was used to calculate the statistical enrichment of DEGs in the KEGG pathway.

### 4.8. GUS Staining of DR5 Reporter Arabidopsis

Seeds of *DR5::GUS* transgenic *Arabidopsis* were germinated and treated with BoPep4 and NaCl as described above. After treatment, histochemical detection of GUS expression was performed as previously described [55]. Briefly, for each independent replicate, 15 *Arabidopsis* seedlings under each treatment were incubated in staining solution in the dark overnight, followed by immersion in 75% ethanol to remove chlorophyll. The experiment was performed with three biological replicates.

### 4.9. Detection of Auxin Contents

Broccoli leaf and root tissues (1.0 g fresh weight) were harvested 7 days after treatments of 200 mM NaCl, 100 nM BoPep4 and the combination of 200 mM NaCl and 100 nM BoPep4. Samples from broccoli seedlings grown in MS liquid medium were collected as a control. Auxins were extracted and quantified using LC-MS/MS for three independent replicates by Nanjing Webiolotech Biotechnology Co., Ltd. (Nanjing, China).

### 4.10. Measurement of Stomatal Aperture

Epidermal peels were isolated from the abaxial surfaces of fully developed *Arabidopsis* leaves and incubated in stomatal-opening solution (50 mM KCl, 0.2 mM CaCl_2_, 10 mM M2-(N-morpholino)-ethane-sulfonic acid (MES)-KOH, pH 6.15) at 23 °C under light for 2 h. The peels were then transferred to MES-KCl buffer containing 20 μM ABA or 1 μM Bopep4 and both for 1 h. The length-to-width ratio was measured by the ImageJ tool [56]. The experiment was performed with three biological replicates.

## 5. Conclusions

In summary, nine plant peptide hormone Pep precursor gene *BoPROPEPs* were identified in the broccoli genome. Salt, ABA, heat, SA and *P. syringae* DC3000 treatments increased the transcriptional levels of *BoPROPEPs* in broccoli sprouts. In particular, the expression level of *BoPROPEP4* was increased most significantly under NaCl treatment. Exogenous application of the synthesized mature peptide BoPep4 enhanced the salt tolerance of broccoli sprouts. Further RNA-seq analysis illustrated that BoPep4 enhanced broccoli’s salt tolerance by regulating auxin and ABA metabolism, as well as cutin and wax biosynthesis. Future studies are needed to uncover the downstream signaling components underlying salt tolerance in broccoli triggered by BoPep4 and BoPEPR receptor complexes.

## Data Availability

The raw RNA-Seq reads were deposited in the National Center for Biotechnology Information BioProject database (http://www.ncbi.nlm.nih.gov/bioproject (accessed on 9 January 2022)) with ID PRJNA795721.

## Supplementary Materials

The following supporting information can be downloaded at: https://www.mdpi.com/article/10.3390/ijms23063090/s1.
