# Supplementary figures and images for: BoPEP4, a C-Terminally Encoded Plant Elicitor Peptide from Broccoli, Plays a Role in Salinity Stress Tolerance
Source: Int J Mol Sci. 2022 Mar 13;23(6):3090. doi: 10.3390/ijms23063090 (PMC8952307; doi:10.3390/ijms23063090)

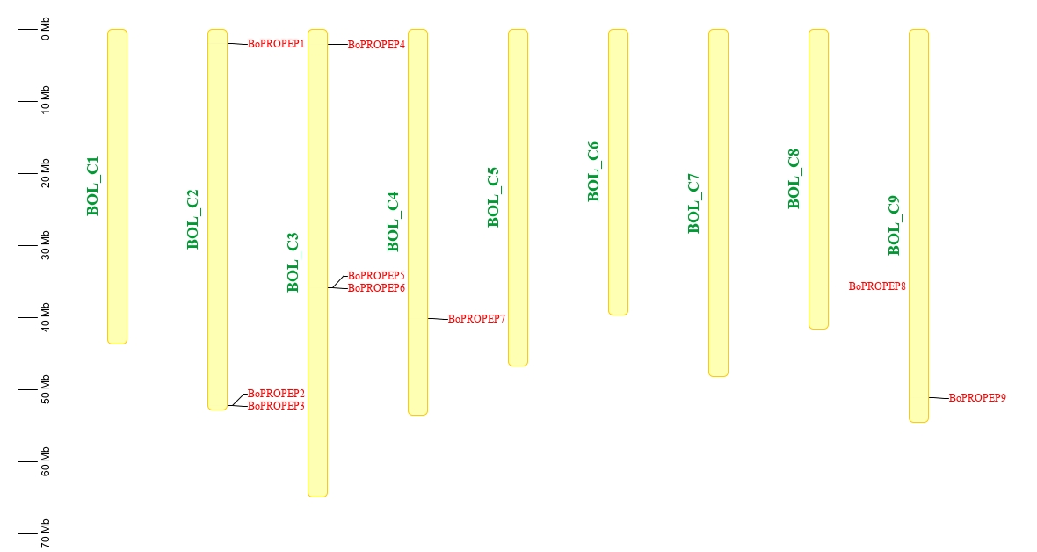

Supplement: Supplementary file 1 [file ijms-23-03090-s001.zip › Fig. S1.jpg]

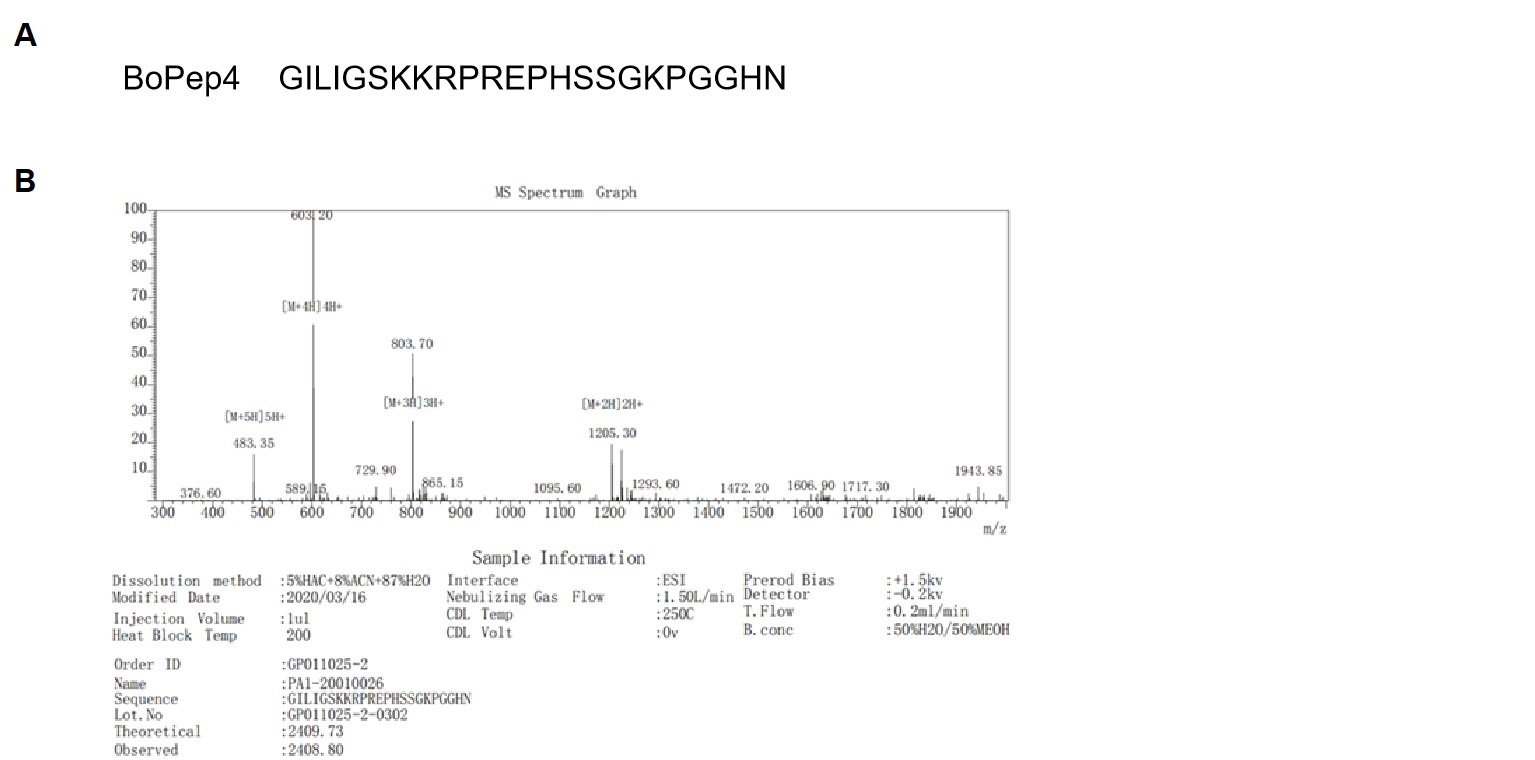

Supplement: Supplementary file 1 [file ijms-23-03090-s001.zip › Fig. S2.jpg]

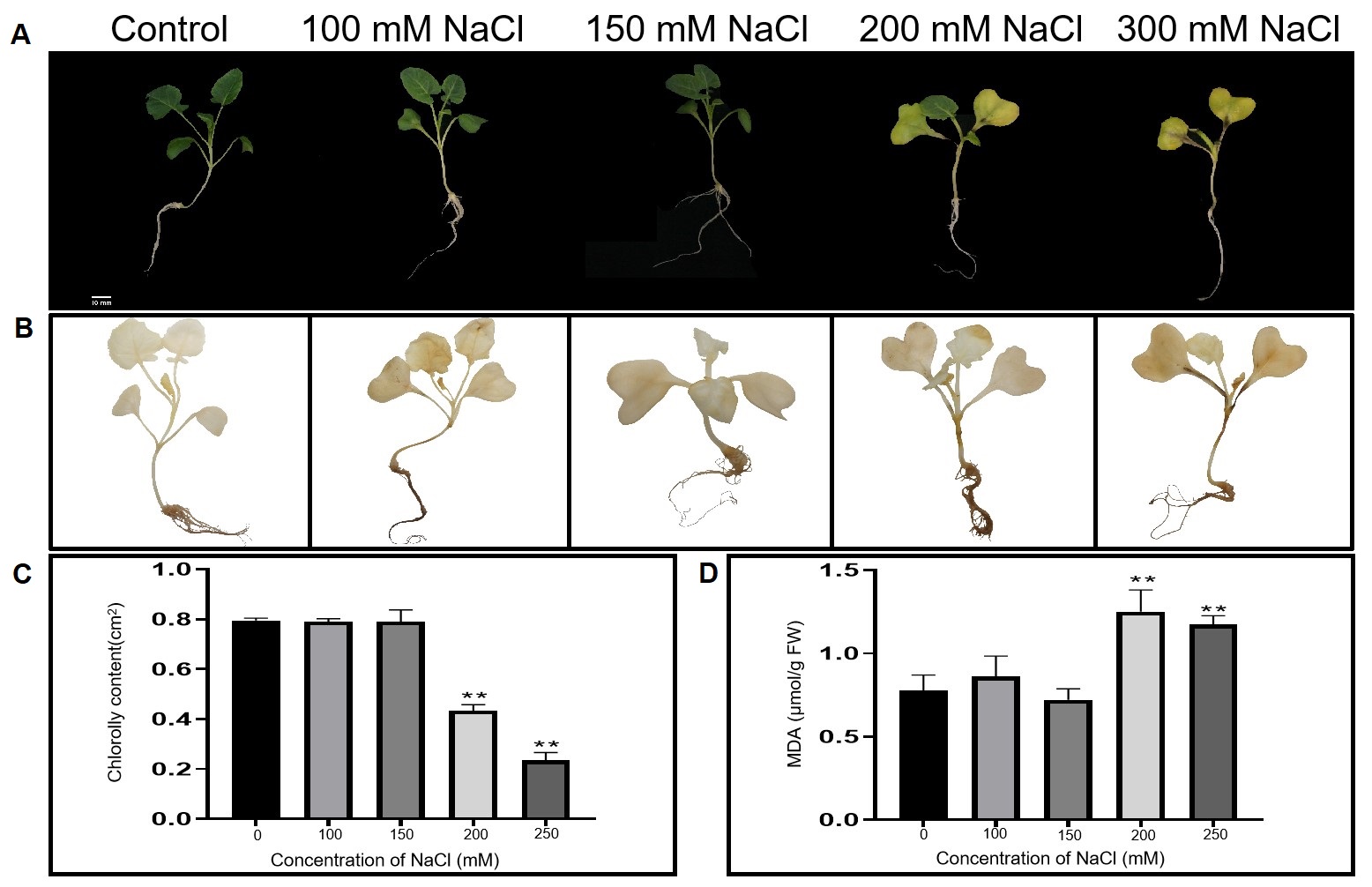

Supplement: Supplementary file 1 [file ijms-23-03090-s001.zip › Fig. S3.jpg]

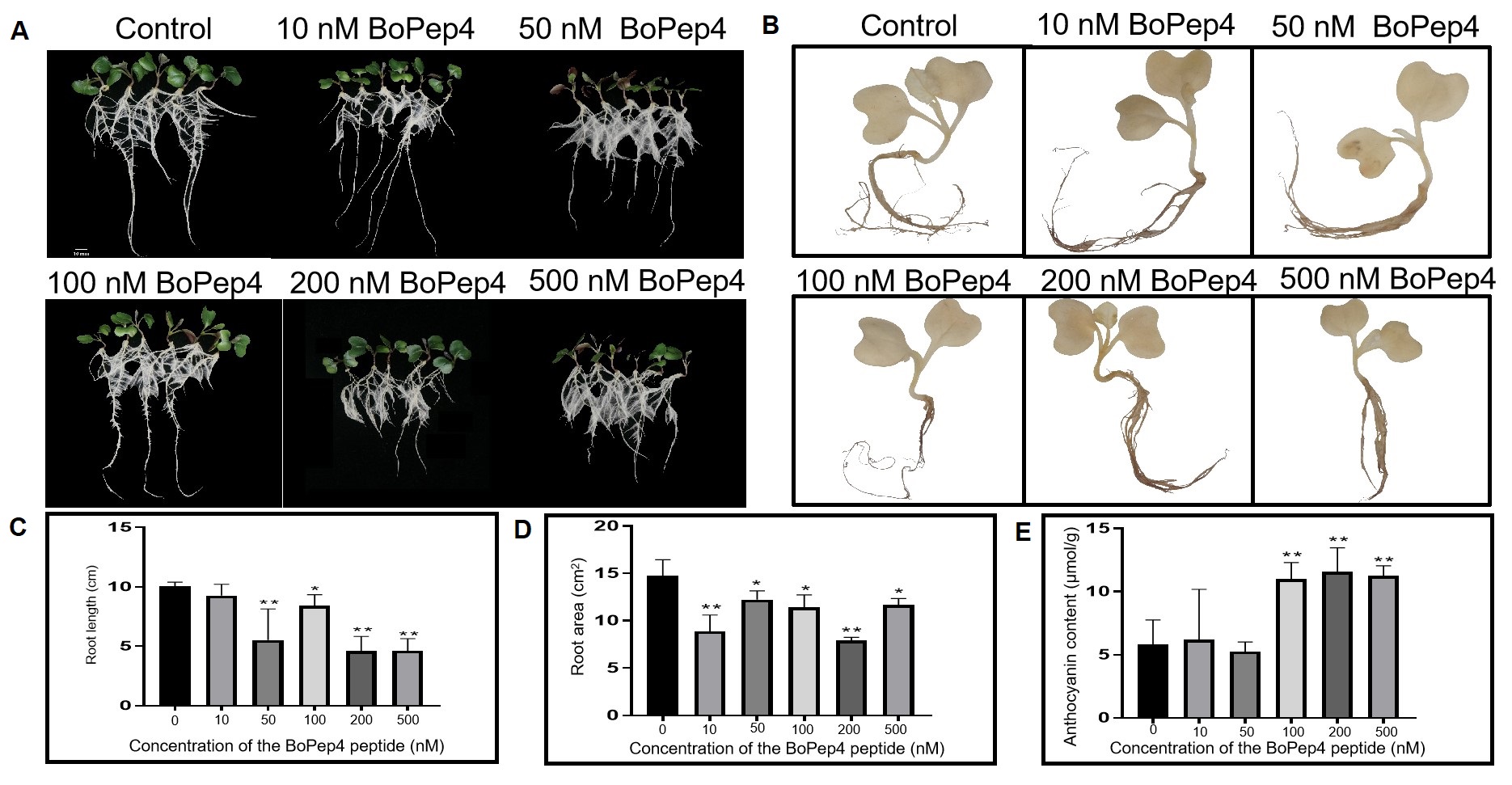

Supplement: Supplementary file 1 [file ijms-23-03090-s001.zip › Fig. S4.jpg]

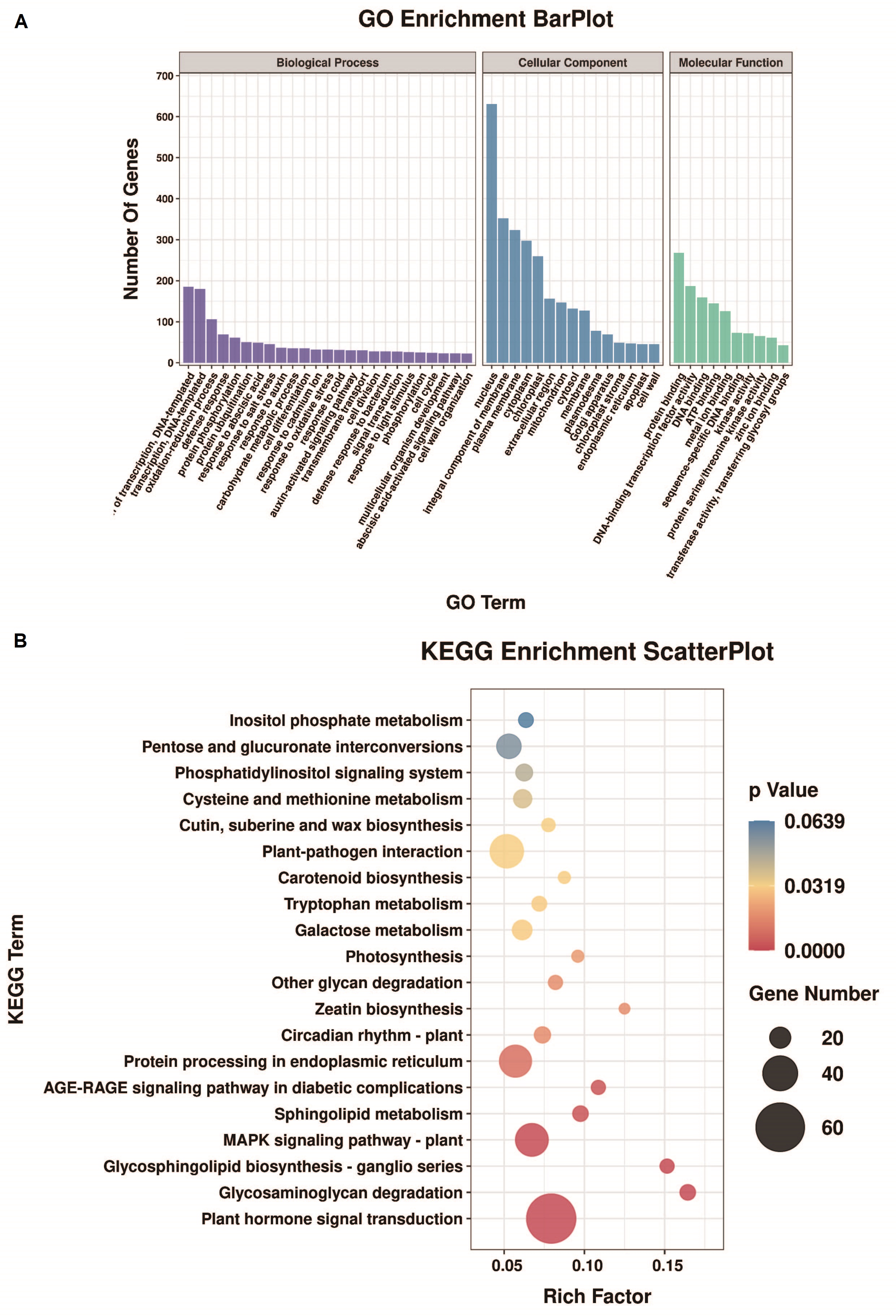

Supplement: Supplementary file 1 [file ijms-23-03090-s001.zip › Fig. S5.jpg]
